# Rapid In Vitro Assessment of Antimicrobial Drug Effect Bridging Clinically Relevant Pharmacokinetics: A Comprehensive Methodology

**DOI:** 10.3390/pharmaceutics15061671

**Published:** 2023-06-07

**Authors:** Michael Nikolaou, Vincent H. Tam

**Affiliations:** 1 Chemical & Biomolecular Engineering Department, University of Houston, Houston, TX 77204, USA; vtam@uh.edu; 2 Department of Pharmacy Practice & Translational Research, University of Houston, Houston, TX 77204, USA

**Keywords:** pharmacokinetics, pharmacodynamics, antimicrobials, antimicrobial resistance, combination therapy, mathematical modeling

## Abstract

Rapid in vitro assessment of antimicrobial drug efficacy under clinically relevant pharmacokinetic conditions is an essential element of both drug development and clinical use. Here, we present a comprehensive overview of a recently developed novel integrated methodology for rapid assessment of such efficacy, particularly against the emergence of resistant bacterial strains, as jointly researched by the authors in recent years. This methodology enables rapid in vitro assessment of the antimicrobial efficacy of single or multiple drugs in combination, following clinically relevant pharmacokinetics. The proposed methodology entails (a) the automated collection of longitudinal time–kill data in an optical-density instrument; (b) the processing of collected time–kill data with the aid of a mathematical model to determine optimal dosing regimens under clinically relevant pharmacokinetics for single or multiple drugs; and (c) in vitro validation of promising dosing regimens in a hollow fiber system. Proof-of-concept of this methodology through a number of in vitro studies is discussed. Future directions for the refinement of optimal data collection and processing are discussed.

## 1. Introduction

Rapid assessment of antimicrobial drug efficacy in clinically relevant pharmacokinetic conditions is fundamental for both new drug development and clinical drug use [1]. This need is particularly pressing for resistant strains, as they pose a serious threat to human health [2]. Assessing the effect of drugs on pathogenic bacteria can be typically measured in vitro through the exposure of a bacterial population to (one or more) drugs at various time-invariant concentrations over a period of time (time–kill experiments). The number of live cells in the bacterial population at distinct points in that time period can be typically assessed by quantitative culture (plating) methods [3]. As widely used as this simple approach is, it provides limited data because plating can realistically be performed only at a few time points during a corresponding experiment (see [4] and references therein). As a result, simple indicators of drug efficacy against bacterial strains, such as the minimum inhibitory concentration (MIC), the area under the curve (AUC), and others, are in widespread use [5,6,7]. Unfortunately, such indicators often fail at predicting therapeutic outcomes under realistic pharmacodynamic and pharmacokinetic conditions [1,8], particularly when a bacterial population comprises subpopulations of varying degrees of resistance [4,9,10,11,12]. More detailed pharmacodynamic models could provide a better picture of drug efficacy [13]. However, developing such models from scant measurements based on plating during short-term time–kill experiments is not practical, if at all feasible.

An obvious way to address this issue would be to collect more measurements of bacterial population size at densely spaced time points during drug exposure. Accomplishing this by plating would be practically infeasible, particularly in situations where time or resources are limited yet reliable results are needed quickly, e.g., in a clinical setting. Among a number of, in principle, feasible options (see Section 4), a long-known, potentially efficient alternative to plating would be to take measurements of sample turbidity (cloudiness) using optical density (OD) methods (spectrophotometry) [14,15,16]. While optical density measurements rely on well-established principles (of light propagation through a liquid) and can easily provide a virtually continuous real-time stream of data in a modern instrument, they have a serious limitation: they *count both live and dead cells* of a bacterial population in a suspension, as both kinds of cells block/absorb light, thus affecting the resulting optical signal. As a result, optical density measurements are routinely taken only in studies focusing on growing populations of cells (e.g., to study contamination) and, until recently, have not been applied to shrinking populations, the very focus of time–kill experiments with bacterial populations exposed to drugs.

This limitation was removed in a series of recent publications that established a novel methodology, which is the main focus of this paper. Specifically, we present here a distilled synthesis of the individual prior results, which collectively constitute the novel integrated methodology just mentioned. This methodology straddles the span from the automatic collection of in vitro longitudinal time–kill data (capturing the drug effect on a bacterial population) to the design and in vitro testing of therapeutic dosing regimens for drugs following clinically relevant pharmacokinetics. While this methodology is still undergoing refinements and enhancements, there is already a body of work at a stage mature enough for direct use, hence worth communicating.

In the rest of the paper, in Section 2, we summarize the basic elements of the proposed methodology referred to above, and in Section 3, we present a selection of basic proof-of-concept outcomes presented in the literature. Finally, we discuss potential improvements and extensions of this methodology.

## 2. Materials and Methods

The essence of the methodology referred to in Section 1 lies in the use of data-driven mathematical modeling tools which extract useful information from longitudinal time–kill data collected by an optical density instrument. Implementation of this methodology entails the following elements:

1. Automated collection of longitudinal optical density measurements of several bacterial cell suspensions by an optical density instrument, each suspension exposed to single or multiple drugs at a time-invariant concentration;
2. Feeding the data collected in the previous step into a mathematical model to estimate the kill rate of the bacterial subpopulation least susceptible to the drug as a function of drug concentration;
3. Use of the drug-concentration-dependent kill rate estimate from the previous step to design dosing regimens predicted to eradicate a bacterial population exposed to a drug following clinically relevant pharmacokinetics;
4. Validation test of promising dosing regimens from the previous step in an in vitro hollow fiber infection model mimicking clinically relevant pharmacokinetics in humans;
5. We elaborate on each of the above elements next.

### 2.1. Longitudinal Optical Density Measurements of Bacterial Cell Suspension under Drug Exposure

**Figure 1:**
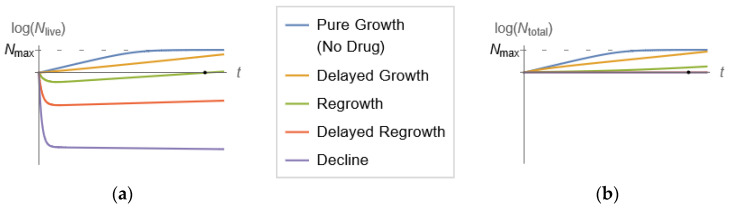
Qualitative patterns of the number of live (**a**) and total (**b**) bacterial cells in suspension ($N_{\mathrm{live}}$ and $N_{\mathrm{total}}$, respectively) exposed to time-invariant antibiotic concentrations in multifold dilutions. The same scale is used for both $N_{\mathrm{live}}$ and $N_{\mathrm{total}}$. Bacterial subpopulations of varying degrees of drug resistance are considered. As drug concentration is set at increasingly higher values, the bacterial population response over time exhibits the five patterns shown. Note that while the number of live cells may decline (**a**), the total number of cells (both live and dead) will never decline (**b**). An optical density instrument can only produce the curves in (**b**). In fact, for high concentrations of the antibiotic, the curves for $N_{\mathrm{total}}$ are practically indistinguishable over time. A central focus of this paper is to summarize a mathematical model-based methodology for obtaining the curves of (**a**) from the curves in (**b**), a task that is arguably impossible based on inspection alone.

In a spectrophotometer, a cell population in suspension is placed in a transparent cuvette, and light is shone on it [17]. Because cells impart turbidity (cloudiness) to the suspension as they absorb and scatter light, the intensity of transmitted light is lower than the intensity of the incident light. Comparing the two intensities provides a quantitative assessment of the number of cells in suspension (optical density). Optical density is roughly proportional to the biomass in the cell suspension in a given range that is specific to the cell type. The simplicity and ability to generate abundant longitudinal data have made spectrophotometry the method of choice for measurements of bacterial *growth* in related applications. The inherent drawback of spectrophotometry, as already mentioned, is its inability to distinguish between live and dead cells, a limitation of utmost importance in time–kill experiments, where bacterial populations are expected to *decline* [18]. Indeed, whereas in bacterial growth experiments, live cells quickly far outnumber dead cells, time–kill experiments with substantial bacterial killing experience the reverse. In fact, bacterial populations with resistant subpopulations may exhibit interesting behavior, as depicted in Figure 1, adapted from [18]. This figure indicates that for successive multiples of drug concentrations, the number of live cells, $N_{\mathrm{live}}$, may qualitatively exhibit pure growth, delayed growth, decline followed by regrowth or delayed regrowth, and finally, continued decline (Figure 1a). However, what optical density measurements will indicate is a set of curves for the total number of cells (comprising both live and dead cells, $N_{\mathrm{total}}$) that never decline, as shown in Figure 1b.

*It is this inherent limitation of optical density instruments that is addressed by the integrated mathematical modeling methodology presented here*, as detailed in subsequent sections. Of course, in addition to the above inherent limitation, secondary issues with optical density instruments may arise from multiple difficulties in the reliable translation of optical density to a number of bacterial cells. Nevertheless, such difficulties, which are expounded on in Section 4, proved surmountable in the work reported here, a fact that underscores the remarkable robustness of the proposed methodology [18,19] and raises expectations for improvements with the future availability of better optical density instruments.

### 2.2. Kill Rate Estimation of Least Susceptible Bacteria as a Function of Drug Concentration

**Figure 2:**
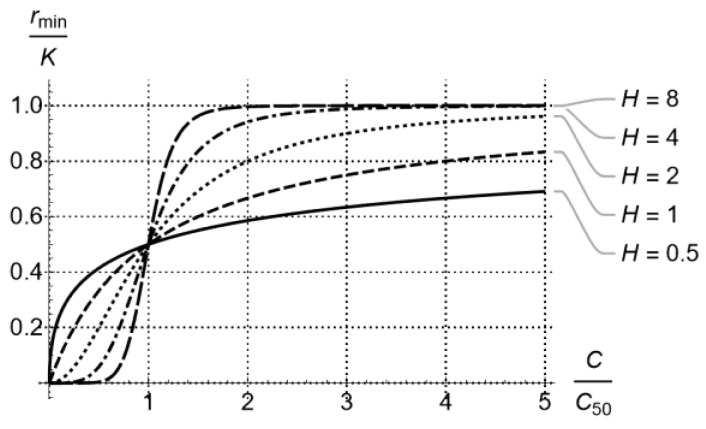
Bactericidal rate of drugs following concentration-dependent (H≪1) or time-dependent (H≫1) activity [1], as captured by the Hill exponent H in Equation (1).

The study of bacterial population dynamics has a long history [20], with a variety of mathematical models used for corresponding quantitative descriptions [13,21]. At the core of these models is the elementary differential equation $dN_{\mathrm{live}}/dt=K_{g}N_{\mathrm{live}}\left(t\right)$ or its counterpart for a saturating bacterial population $dN_{\mathrm{live}}/dt=K_{g}N_{\mathrm{live}}\left(t\right)\left(1-N_{\mathrm{live}}/N_{\max}\right)$, where $N_{\mathrm{live}}$ is the number of live bacterial cells in a population, $K_{g}$ is the physiological growth rate, and $N_{\max}$ is the upper bound of the growing bacterial population [22]. Exposure to bactericidal drugs adds a killing term, $rN_{\mathrm{live}}$, to the right-hand side of the preceding equations, where r is the specific kill rate, dependent on drug concentration C. A typical expression for the specific kill rate is

(1) $$r=K\frac{C^{H}}{C^{H}+C_{50}^{H}}$$

where H is the Hill exponent of sigmoidicity [9,23,24,25] as shown in Figure 2.

For typical time–kill experiments with time-invariant C, solution of the differential equation $dN_{\mathrm{live}}/dt=\left(K_{g}-r\right)N_{\mathrm{live}}\left(t\right)$ is $N_{\mathrm{live}}\left(t\right)=N_{\mathrm{live}}\left(0\right)\exp\left[\left(K_{g}-r\right)t\right]$, which corresponds to a straight line of slope $K_{g}-r$ in a plot of $\log\left(N_{\mathrm{live}}\left(t\right)\right)$ vs. t.

As conceptually useful as such linear plots are, most practical situations of bacterial populations exposed to drugs involve subpopulations of varying susceptibility to the drug(s), corresponding to a distribution of values of r≥0 over a bacterial population at any given drug concentration C [9,10]. The result is curved rather than straight lines for $\log\left(N_{\mathrm{live}}\left(t\right)\right)$, as depicted, for example, in Figure 1a. For such cases, it was shown [11,12] that the size $N_{\mathrm{live}}(t)$ of a heterogeneous bacterial population exposed to one or more drugs at time-invariant concentration C is well captured by the equation

(2) $$\begin{array}{c} \ln\left[\frac{N_{live}\left(t\right)}{N_{0}}\right]=\left(K_{g}-r_{min}\right)t+\lambda \left(e^{-at}-1\right)-\\ -\ln\left[1+K_{g}\frac{N_{live}\left(0\right)}{N_{max}}\int_{0}^{t}\exp\left[\left(K_{g}-r_{min}\right)\tau +\lambda \left(e^{-a\tau }-1\right)\right]d\tau \right] \end{array}$$

and the kill rate average and variance over time are well captured by the equations

(3) $$\begin{array}{c} \mu \left(t\right)=r_{min}+\left(\mu \left(0\right)-r_{min}\right)\exp\left[-\frac{\mu \left(0\right)-r_{min}}{\lambda }t\right]=\\ =r_{min}+\lambda ae^{-at} \end{array}$$

(4) $$\begin{array}{c} {\sigma (t)}^{2}=\frac{{\left(\mu \left(0\right)-r_{min}\right)}^{2}}{\lambda }exp\left[-\frac{\mu (0)-r_{min}}{\lambda }t\right]=\\ =\lambda a^{2}e^{-at} \end{array}$$

where:

$N_{live}\left(t\right)$ is the live bacterial population size with an initial value of $N_{0}$;
$K_{g}$ is the physiological net growth rate of the entire bacterial population, common for all subpopulations;
$r_{min}$ is the kill rate induced by the antibiotic on the most resistant (least susceptible) subpopulation;
$N_{max}$ is the maximum size of a bacterial population reaching saturation under growth conditions;
$\mu \left(t\right)$ is the kill rate average over the bacterial population at time t;
$\sigma {\left(t\right)}^{2}$ is the kill rate variance over the bacterial population at time t.

λ>0,a>0 are constants associated with the initial decline of the average kill rate of the population and correspond to the Poisson distributed variable ($r-r_{min})/a$ with average and variance equal to λ. Note that no assumptions about the mechanisms that confer bacterial resistance have been made to derive the above Equations (2)–(4).

The parameters $K_{g},r_{min},\lambda ,a,N_{max}$ that appear in Equation (2) can be estimated by regression, *assuming enough values of* $N_{\mathrm{live}}(t)$ *are available*. The preceding statement immediately justifies why plating methods for measurement of $N_{\mathrm{live}}(t)$ are impractical for estimation of $K_{g},r_{min},\lambda ,a,N_{max}$: at least 10–20 data points would be needed for reasonable parameter estimates [9,10].

This limitation, posed by plating-based measurements, is overcome by the optical density-based methods discussed, which can routinely generate measurements every minute or so. However, as already mentioned, their measurements are of $N_{\mathrm{total}}$ rather than of $N_{\mathrm{live}}$. To make measurements of $N_{\mathrm{total}}$ usable in parameter estimation, the following equation was derived [18]:

(5) $$\begin{array}{c} \frac{N_{total}\left(t\right)}{N_{0}}=e^{\lambda \left(e^{-at}-1\right)+\left(K_{g}-r_{min}\right)t}+\\ +e^{-\lambda }\lambda^{\frac{K_{g}-r_{min}}{a}}\left(\frac{K_{d}+r_{min}}{a}\int_{\lambda e^{-at}}^{\lambda }z^{-1+\frac{r_{min}-K_{g}}{a}}e^{z}dz+\int_{\lambda e^{-at}}^{\lambda }z^{\frac{r_{min}-K_{g}}{a}}e^{z}dz\right) \end{array}$$

when $N_{0}\ll N_{\max}$, which is typical for time–kill experiments. (The full expression for $N_{0}$ not far from $N_{\max}$ is also shown in [18]). In fact, it may be numerically simpler and conceptually insightful to use the following two differential equations (from which Equation (5) is derived for $N_{0}/N_{\max}\approx 0$) in parameter estimation based on measurements of $N_{\mathrm{total}}\left(t\right)$:

(6) $$\frac{dN_{live}}{dt}=\left(-K_{g}\frac{N_{live}\left(t\right)}{N_{max}}+K_{g}-r_{min}-\lambda ae^{-at}\right)N_{live}\left(t\right)\approx \left(K_{g}-r_{min}-\lambda ae^{-at}\right)N_{live}\left(t\right)$$

(7) $$\frac{dN_{total}}{dt}=\left(K_{g}\left[1-\frac{N_{live}\left(t\right)}{N_{max}}\right]+K_{d}\right)N_{live}\left(t\right)\approx \left(K_{g}+K_{d}\right)N_{live}\left(t\right)$$

where $K_{d}$ is the natural death rate of bacterial cells.

Equations (6) and (7) shed light on the nature of the parameter estimation problem:

First, estimates of $K_{g},K_{d}$ can be obtained from a simple time-growth experiment (C=0) for which there is no drug-induced kill rate, i.e., $r_{\min}=0$ and λ=0, by default. Then, estimates of $N_{\mathrm{live}}\left(t\right)$ are reasonably well obtained from Equation (7), provided that $dN_{\mathrm{total}}/dt$ can be estimated with reasonable accuracy. It is for this purpose that *optical density-based measurements* of $N_{\mathrm{total}}(t)$ *at closely spaced points in time become crucial*, as they allow a reasonably accurate estimation of $dN_{\mathrm{total}}/dt$, hence of $N_{\mathrm{live}}\left(t\right)$ via Equation (7), and finally, the parameters $r_{min},\lambda ,a$ via standard regression using Equation (6).

**Figure 3:**
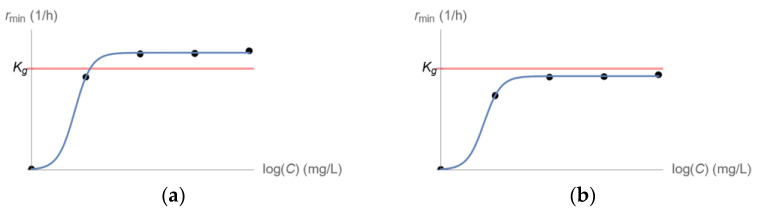
Fit of Equation (1) to values of $r_{\min}$ estimated from time–kill experiments with a bacterial population exposed to distinct drug concentrations C. (**a**) Eradication of the entire bacterial population is predicted for high enough drug concentrations C. (**b**) No drug concentration C can eliminate the entire bacterial population.

Of the parameters estimated via the exercise just described, the one *critical for dosing regimen design* is $r_{\min}$, which is the kill rate of the least susceptible (most resistant) subpopulation. (Note that in long enough time–kill experiments, Equation (3) immediately suggests that the average kill rate, $\mu \left(t\right)$, quickly tends to $r_{\min}$). The importance of $r_{\min}$ stems from the fact that complete eradication of a bacterial population exposed to a drug at concentration C occurs if and only if

(8) $$r_{\min}>K_{g}$$

i.e., *the kill rate of the least susceptible bacterial subpopulation should be greater than the corresponding population growth rate* (see Equation (5) and discussion in [18]). The dependence of $r_{\min}$ on C is typically considered to follow the form of Equation (1), as shown qualitatively in Figure 3 (two instances of the general Figure 2), which qualitatively depicts a fit of Equation (1) to values of $r_{\min}$ estimated from time–kill experiments at distinct drug concentrations C. It is noted that counterparts of Equation (1) can be fit to effective concentrations concerning multiple drugs, but this pharmacodynamics issue [26,27,28] is beyond the scope of this paper and will be explored elsewhere. The outcome of fitting $r_{\min}$ to C is crucial for designing effective dosing regimens under clinically relevant pharmacokinetics, as will be discussed in the next section.

### 2.3. Dosing Regimen Design for Bacterial Eradication under Clinically Relevant Pharmacokinetics

The preceding discussion in Section 2.1 and Section 2.2 is concerned with pharmacodynamics (PD), i.e., the bactericidal effect of a drug at a certain concentration. This knowledge is one of the two fundamental components of pharmacology for antimicrobial therapy [1]. The second fundamental component is pharmacokinetics (PK), i.e., the absorption, distribution, and metabolism/elimination of drugs. In this section, we describe how pharmacodynamic information, as acquired by the approach described in the previous two sections, can be combined with related pharmacokinetics for design of effective drug dosing regimens.

**Figure 4:**
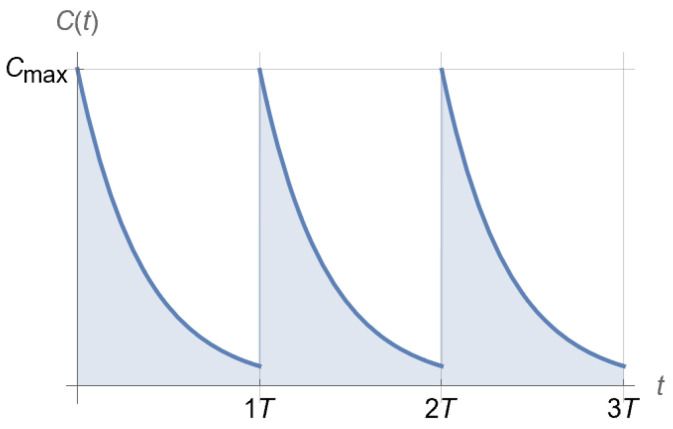
Clinically relevant pharmacokinetics of drug injection every T time units at peak concentration $C_{\max}$ for a drug with a corresponding half-life $t_{1/2}$.

A drug can certainly be clinically administered in a number of ways, each resulting in a corresponding drug concentration profile in a patient over time—from time-varying to time-invariant. In addition to variability over time, administered drug concentration in a patient typically exhibits topical variability as well, following corresponding pharmacokinetics. For example, different profiles typically arise between plasma (blood) and tissue concentration, as there are multiple compartments from an injection point to targeted tissues, and the corresponding dynamics for each compartment are different for different drugs [29]. A simple pharmacokinetic profile for periodic drug injection and subsequent first-order elimination of the drug is shown in Figure 4, where the drug concentration $C\left(t\right)$ is plotted over time t. This choice lends itself to both simple theoretical analysis and experimental testing in an in vitro hollow-fiber infection model (Section 2.4 below). Alternatives are expounded on in Section 4.

**Figure 5:**
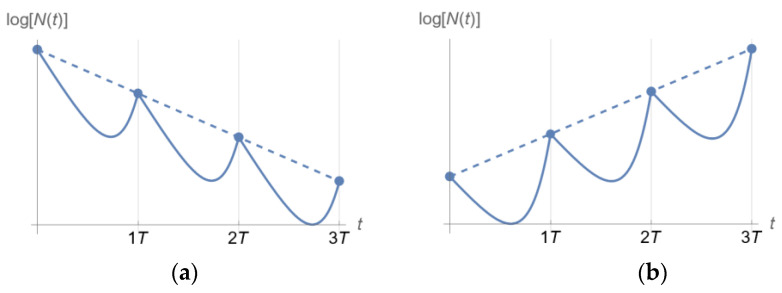
Qualitatively distinct outcomes of a homogeneous bacterial population exposed to a drug following the PK in Figure 4, depending on $D/K_{g}$ (Equation (9)). (**a**) Bacterial population decline for $D/K_{g}>1$. (**b**) Bacterial population growth for $D/K_{g}<1$.

The profile of Figure 4 may produce one of the two outcomes shown in Figure 5 for a homogeneous microbial population [30].

It can be shown [30] that outcome (a) of Figure 5, i.e., elimination of a bacterial population by drug administration following the PK profile of Figure 4, is predicted if and only if

(9) $$D≝\frac{1}{T}\int_{0}^{T}r_{\min}\left(C\left(\eta \right)\right)d\eta >K_{g}$$

for the least susceptible bacterial subpopulation.

**Figure 6:**
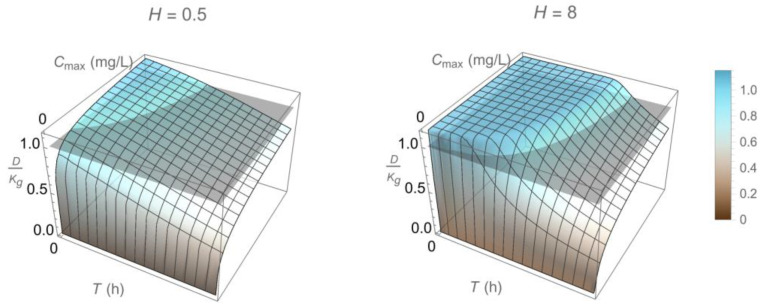
Qualitative dependence of $D/K_{g}$ (compared to 1, gray plane) on design parameters T and $C_{\max}$ (period of injection and peak drug concentration, respectively) associated with a dosing regimen following the pharmacokinetic pattern of Figure 4 for a drug of corresponding half-life and two different values of the Hill factor H (0.5 vs. 8, Figure 2).

Equation (9) is the main outcome of this section and can be used to *complete a dosing regimen design* that ensures, with confidence, that $D>K_{g}$. It provides a direct link between PK and PD, as drugs with the same PD but different PK or with the same PK and different PD will generally result in different D, hence will require different dosing regimens to achieve similar bactericidal outcomes. A detailed investigation of this aspect is provided in [30] and, in a broader context, in [4]. Qualitatively, what one can expect when selecting $C_{\max}$ and T for a dosing regimen accommodating the PK of a drug with a half-life $t_{1/2}$ (Figure 4) depends on all three parameters $K,C_{50},$ and H of $r_{\min}$ (see Equation (1)). Of these parameters, H determines how sigmoidal $r_{\min}$ is (Figure 2) and, ultimately, whether the drug exhibits time-dependent or concentration-dependent behavior [1]. The resulting $D/K_{g}$ qualitatively follows the patterns shown in Figure 6, which provides a link between PK and PD for corresponding drugs.

### 2.4. Validation Test of Promising Dosing Regimens in an In Vitro Hollow Fiber Infection Model

**Figure 7:**
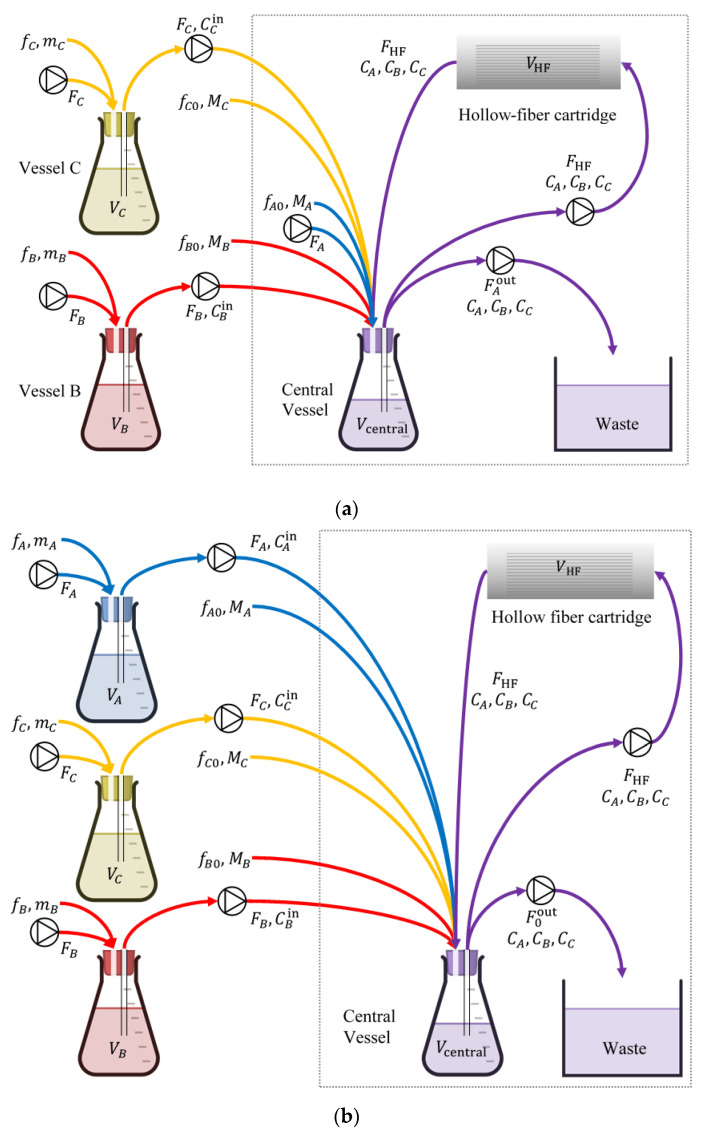
In vitro hollow fiber infection model (HFIM) for testing the effectiveness of three drugs that follow different pharmacokinetics. Design parameters shown take values according to the formulas developed in [44]. (**a**) In-series configuration: adds vessels B, C to the basic central vessel of the single-drug design. (**b**) In-parallel configuration: adds vessels A, B, C to the basic central vessel of the single-drug design, offering additional flexibility on the range of pharmacokinetics to be simulated in vitro. The framed areas in both (**a**,**b**) refer to the basic design for a single drug. Each framed area, along with vessel B and associated streams, corresponds to Blaser’s long-known configuration [31,36] for two drugs.

While the preceding methodology can use optical density measurements from time–kill experiments to rapidly design dosing regimens expected to be effective under realistic pharmacokinetics, there is inherent uncertainty in such expectations [26,27,28]. In vitro testing in a hollow-fiber infection model (HFIM, Figure 7) [31,32] can be used to test whether expected outcomes under realistic pharmacokinetics will indeed be achieved [33,34,35]. Simple HFIM designs for a single drug (in terms of both structure and design parameter values) were heuristically extended in the 1980s to the designs of two drugs with different pharmacokinetics [31,36]. However, recent developments in the treatment of multidrug-resistant bacterial infections [37,38,39,40,41] have made it necessary to test three or even more drugs in an HFIM before a corresponding combination is put to clinical use [26,42,43]. This made it necessary to advance the state of the art in HFIM design. A comprehensive new design method was developed in [44], where (a) new configurations beyond the standard in-series configuration of [31,36] were developed (Figure 7), (b) explicit formulas were derived for setting design parameters to values that result in desired pharmacokinetics for each drug in the hollow fiber cartridge, and (c) even for two drugs, an entire family of new designs beyond Blaser’s [36] was provided. In addition to making designs for three or more drugs feasible, these advancements substantially improved flexibility for the satisfaction of design objectives and constraints in an HFIM and paved the way for further extensions concerning various pharmacokinetic profiles (see Section 4).

## 3. Results

### 3.1. Longitudinal Optical Density Measurements of Bacterial Cell Suspension under Drug Exposure

Proof of concept was provided in [18] for the information extraction capabilities of model-based analysis of longitudinal optical density measurements from a bacterial suspension exposed to antibiotics in time–kill experiments, as outlined in Section 2.1. In [18], levofloxacin in fourfold dilutions was used against *Acinetobacter baumannii,* ATCC BAA747. In that study, longitudinal measurements of the bacterial population size (of the kind shown in Figure 1b) for both live and dead cells were collected by an optical density instrument (BacterioScan model 216Dx) at the early stages of its development at the time. Even though the instrument could not explicitly report declining population patterns for live cells, as explained in Section 2.1, the model-based analysis of optical density data for both live and dead cells could reconstruct the live cell population over time with remarkable fidelity, correctly estimating live population decline and subsequent long-term regrowth patterns from data of as little duration as six hours. These patterns were confirmed with separate, far more time-consuming, plating-based measurements in the same study, validating the credibility of the method and suggesting its potential use in dosing regimen design for clinically relevant pharmacokinetics, as discussed next.

### 3.2. From Optical Density Measurements to Dosing Regimen Design for Clinically Relevant Pharmacokinetics

**Figure 8:**
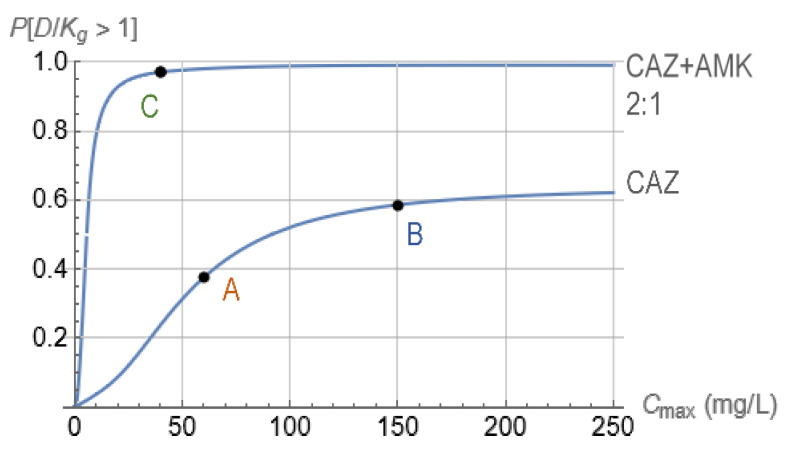
Probability of satisfaction of Equation (9) for bacterial exposure to ceftazidime (CAZ) and combination of ceftazidime and amikacin at a mass ratio of 2:1 (CAZ + AMK, 2:1). In both cases, drugs were injected every T=8 h with subsequent exponential decline of each concentration from its peak of $C_{\max}$, corresponding to half-life of $t_{1/2}=2.5\;h$. $C_{\max}=60\mathrm{or}150\mathrm{mg}/L$ for points A and B, respectively, and $C_{\max,\;\mathrm{ceftazidime}}/C_{\max,\;\mathrm{amikacin}}=40/20\;(\mathrm{mg}/L)/(\mathrm{mg}/L)$ for point C were selected for testing in the in vitro HFIM with corresponding probabilities for $D\backslash K_{g}>1$ equal to 59%, 38%, and 98%, respectively.

The capability of the proposed model-based methodology to start from optical density time–kill measurements and eventually guide individualized dosing regimen design for clinically relevant pharmacokinetics was experimentally validated in vitro in [19]. In that study, longitudinal optical density measurements were collected using the same instrument as in Section 3.1. In corresponding time–kill experiments, *Acinetobacter baumannii*, ATCC BAA747, was exposed to fourfold dilutions of (a) ceftazidime and of (b) ceftazidime/amikacin in a concentrations ratio of 2:1. Model-based data analysis was performed for both cases, considering periodic injections every T=8 h with subsequent exponential drug decline corresponding to a half-life of $t_{1/2}=2.5\;h$. The design parameter left to choose from for both cases was $C_{\max}$, the peak concentration of ceftazidime (Figure 4). The outcome of the model-based analysis is summarized in Figure 8. In that figure, it is indicated that ceftazidime alone is not predicted with a high likelihood to suppress the entire bacterial population, even at high concentrations (Figure 8, CAZ). By contrast, the ceftazidime/amikacin 2:1 combination is predicted in the same figure (CAZ + AMK, 2:1) to have a very high likelihood of suppressing the bacterial population, even at relatively modest concentrations.

**Figure 9:**
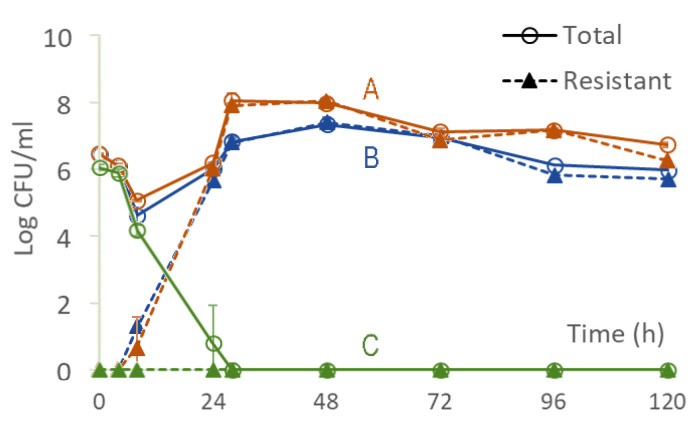
Bacterial response in the hollow fiber infection model corresponding to points A, B, and C of Figure 8. The responses associated with points A and B (orange and blue) correspond to injecting ceftazidime equivalent to 1 g ($C_{\max}=60\mathrm{mg}/L$) and 2.5 g ($C_{\max}=150\mathrm{mg}/L$) respectively, administered every 8 h, as shown in Figure 4. The response associated with point C (green) corresponds to injecting a combination of ceftazidime and amikacin (2:1 mass ratio) with ceftazidime equivalent to 0.67 g and amikacin equivalent to 5mg/kg administered every 8 h, as shown in Figure 4. Data are shown as mean ± standard deviation.

The predictions of Figure 8 were experimentally tested by implementing the antibiotic profile of Figure 4 in an in vitro hollow-fiber infection model, using $C_{\max}=60\mathrm{or}150\mathrm{mg}/L$ for points A and B, respectively, and $C_{\max,\;\mathrm{ceftazidime}}/C_{\max,\;\mathrm{amikacin}}=40/20\;(\mathrm{mg}/L)/(\mathrm{mg}/L)$ for point C of Figure 8. The outcomes are shown in Figure 9, where confirmation of predictions established in Figure 8 is evident.

## 4. Discussion

### 4.1. Optical Density Measurements and Alternatives

As already discussed in Section 1, a number of alternatives exist for measuring the number of cells in a population. These include quantitative polymerase chain reaction (PCR) [45,46], fluorescence microscopy [47,48], enzyme-linked immunosorbent assay (ELISA) [49], fluorescence-based microplate reader [50,51,52], flow cytometry [47,53,54], fiber-based fluorescence spectroscopy [55]), and microcalorimetry [56]. While they all have a significant role to play in corresponding applications, they also have disadvantages related to complexity or the current state of development, thus making optical density methods a viable option for the purposes of this study. A recent, informative review [57] summarizes such methods and their corresponding applications. For all methods, improvements in both instrument hardware and software are reasonable to anticipate in the future.

For optical density instruments, improvements would be welcome to address sources of systematic errors, such as the following: To extend their dynamic range, many optical density instruments change the measurement method from scattering to absorption based on a threshold value of bacterial concentration, thus possibly introducing calibration errors [19]. Dead cells decompose over time, thus changing the optical signature of the cell suspension and making corresponding adjustments necessary. The concentration of cells in suspension used in the instrument may not be uniform if mixing is not adequate, thus biasing measurements. A number of antibiotics, such as fluoroquinolones, can induce morphological changes in exposed bacteria (e.g., filaments at concentrations close to the MIC), thus inadvertently changing again the optical signature observed.

While the results presented here demonstrate the value of the proposed methodology, improvements in optical density instruments are anticipated to increase the methodology’s utility.

### 4.2. Clinically Relevant Pharmacokinetics and Design and In Vitro Testing of Dosing Regimens

A confident estimate of the effect of drug concentration, C, on the specific kill rate, $r_{\min}$, of the least susceptible bacterial subpopulation via Equation (1) (as discussed in Section 2.2) immediately allows for the use of Equation (9) to design of an effective dosing regimen that accommodates specified pharmacokinetics. Indeed, regardless of the particular form of $C\left(t\right)$ in Equation (9), the effectiveness of a dosing regimen rests on whether $D>K_{g}$. While the preceding analysis was carried out for the profile of $C\left(t\right)$ shown in Figure 4, other profiles, such as profiles corresponding to multi-compartment models, may easily be considered [29].

It should also be noted that while the in vitro testing of dosing regimens in an HFIM outlined in Section 2.4 is specifically designed for pharmacokinetics, as shown in Figure 4, methods for experimental designs that address pharmacokinetics that correspond to multi-compartment models are directly feasible, using the Laplace-transforms-based method described in [44]. This is left for future investigation.

## 5. Conclusions and Future Work

The results presented here highlight the potential for clinicians to use the proposed method for individualized dosing regimen design. The utility of the proposed method can be further consolidated and expanded with additional future work. Potential items to study include the following:

- Instrumentation improvements that may improve the quality of the data (reduction of systematic error);
- A wider range of clonally diverse bacteria;
- Bacteria with various resistance mechanisms;
- Different antibiotics, particularly with pharmacodynamics and pharmacokinetic differences;
- Combination therapy, particularly the interplay between pharmacodynamics and pharmacokinetics;
- Automation of computations through software development;
- Testing of in vivo relevance in animal infection models.

## 6. Patents

A US patent application has been filed for the method presented here [58].

## Data Availability

Not applicable.
